# ﻿Three new species of *Atkinsoniella* (Hemiptera, Cicadellidae, Cicadellinae) from southwestern China

**DOI:** 10.3897/zookeys.1221.129125

**Published:** 2024-12-31

**Authors:** Yan Jiang, Xiao-Fei Yu, Mao-Fa Yang

**Affiliations:** 1 Institute of Entomology, Guizhou Provincial Key Laboratory for Agricultural Pest Management of the Mountainous Region, Guizhou University, Guiyang 550025, China; 2 Department of Pharmacy, Guizhou Provincial Engineering Research Center of Medical Resourceful Healthcare Products, Guiyang Healthcare Vocational University, Guiyang 550000, China; 3 College of Tobacco Sciences, Guizhou University, Guiyang 550025, China

**Keywords:** Auchenorrhyncha, China, leafhopper, morphology, taxonomy

## Abstract

Three new species of the genus *Atkinsoniella* (Hemiptera: Cicadellidae: Cicadellinae), *A.chongqingana* Jiang & Yang, *A.likuni* Jiang & Yang and *A.biostiolum* Jiang & Yang, **sp. nov.**, collected from southwestern China, are described and illustrated. The two new species, *A.chongqingana* Jiang & Yang, **sp. nov.** and *A.likuni* Jiang & Yang, **sp. nov.**, are similar to *A.nigrominiatula* (Jacobi, 1944), *A.latior* Young, 1986, *A.limba* Kuoh, 1991, *A.dormana* Li, 1992, *A.divaricata* Yang, Meng & Li, 2017, *A.peaka* Yang, Meng & Li, 2017, and *A.zizhongi* Jiang & Yang, 2022 in appearances, but can be distinguished from these species by the characteristic of aedeagus, paraphysis, and pygofer. *Atkinsonellabiostiolum* Jiang & Yang, **sp. nov.** can be distinguished from all the known *Atkinsoniella* species by its special color and markings, as well as males having one ostiole in the center of the base of each subgenital plate. A key to *Atkinsoniella* species from China is provided.

## ﻿Introduction

Southwestern China includes Sichuan Province, Guizhou Province, Yunnan Province, Tibet Autonomous Region, and Chongqing Municipality. Due to its complex topography characterized by significant variations in altitude and numerous mountainous basins, many insects, including Cicadellidae, are rich in biodiversity in Southwest China. Of the 102 valid known species of the genus *Atkinsoniella*, 92 occur in China and distributed in 20 provincial administrative regions (Feng and Zhang 2015; Yang et al. 2017; Naveed and Zhang 2018; Jiang et al. 2022, 2023). Of the 92 known Chinese *Atkinsoniella* species, 72 species are distributed in Yunnan Province, 26 species in Guizhou Province, 21 species in Tibet Autonomous Region, 20 species in Sichuan Province, and 17 species are distributed in Chongqing Municipality (Yang et al. 2017; Jiang et al. 2023). In this study, the descriptions, male genitalia, and habitus photographs of three new species, *Atkinsoniellachongqingana* Jiang & Yang, sp. nov., *A.likuni* Jiang & Yang, sp. nov. and *A.biostiolum* Jiang & Yang, sp. nov. from southwestern China are provided with a key to all Chinese species.

## ﻿Materials and methods

The specimens were collected by sweeping (27–35 sweeps per collecting event) on shrubs and weeds using 2.5 m insect sweep nets (200 mesh) in daylight, and at sunset using a 500W high-pressure mercury lamps; all materials were preserved in absolute ethanol and stored at -20 °C in the laboratory. The abdomens of specimens were detached and soaked in 10% NaOH solution, boiled for ~ 3 min, rinsed with water to remove traces of NaOH, and transferred to glycerol for further dissection, photography, and eventually preserved in PCR tubes with glycerol. The habitus and male genitalia were photographed using a KEYENCE VHX-6000 digital camera and a Nikon Eclipse Ni-E microscope, respectively. Adobe Photoshop 2020 was used to edit compiled images. The length of the body was measured from the vertex to the rear of the forewings using a KEYENCE VHX-6000 digital camera. The morphological terminology is adapted from Young (1968, 1986) and Yang et al. (2017). The holotype and paratypes were deposited at the Institute of Entomology, Guizhou University, Guiyang, China (**GUGC**).

## ﻿Taxonomy

### ﻿Genus Atkinsoniella

Taxon classificationAnimaliaHemipteraCicadellidae

Distant, 1908

7F75BF5E-E01A-5638-82FF-662AD3AF5A46

Atkinsoniella Distant, 1908: 235.
Soibanga Distant, 1908: 236.
Curvufacies Kuoh, 1993: 38.

#### Type species.

*Atkinsonielladecisa* Distant, 1908, type locality India.

#### Distribution.

Palearctic, Oriental.

#### Note.

The comparison of male genitalia morphological characteristics of the nine similar *Atkinsoniella* species is provided in Table 1.

**Table 1.** Comparison of male genitalia morphological characteristics of the nine similar *Atkinsoniella* species.

Species	Pygofer	Pygofer process	Aedeagus	Paraphysis	Style
***A.nigrominiatula* (Jacobi, 1944)**	Posterior portion slightly widened, dorsal margin straight.	Posterior 1/2 tapered, tip acute and not exceeding posterior margin of pygofer.	Entirely short, dorsal margin nearly straight.	Paraphysis with tip tapered and bent dorsad, articulating with aedeagus apically.	Y-shaped
***A.latior* Young, 1986**	Nearly rectangular with posterior margin broadly rounded.	Pygofer process extending posterodorsally, then posteriorly, attenuated and sharply curved apically.	Aedeagus wide, dorsal margin straight, apical part narrower.	Paraphysis pygofer process extending posterodorsally, then posteriorly, attenuated, and sharply curved apically.	Y-shaped
***A.limba* Kuoh, 1991**	Dorsal margin with 1 angular flat process near base.	Pygofer process arising basiventrally and tapered posteriorly.	Aedeagus slender and posterior portion bent dorsad.	Paraphysis with laterally produced flattened part subapically.	Y-shaped
***A.dormana* Li, 1992**	Dorsal margin with 1 angular flat process near base.	Pygofer process bent posterodorsally from median, tip acute.	Aedeagus wide basally, median with pair of triangular flat processes, tip bent dorsad.	Paraphysis with tip tapered and bent dorsad, articulating with aedeagus apically.	Nearly V-shaped
***A.divaricata* Yang, Meng & Li, 2017**	Posterior margin broadly rounded, basal 1/3 of dorsal margin convex and with several macrosetae.	Posterior 1/2 bending dorsad, tip acute.	Base wide, proximal portion slightly curved dorsad.	Medially with wrinkle in ventral view, tip forked and clamped median of aedeagus.	V-shaped
***A.peaka* Yang, Meng & Li, 2017**	Medially bulging outwards, tip sharply flattened and contracted into rounded protrusion, resembling peak of peaked cap.	Base with several microsetae, posterior portion acute and extending straight, tip not reaching posterior margin of pygofer.	Basal 1/3 bent dorsad, medial 1/3 portion approaching paraphysis, tip rounded.	Tip hooked and articulated with proximal aedeagus apically.	V-shaped
***A.zizhongi* Jiang & Yang, 2022**	Posterior portion broadly rounded and bent dorsally.	Arising basiventrally and extending dorsolateral posteriorly of pygofer, apex with transparent membrane dorsad and exceeding posterior margin of pygofer.	Base and tip concave, ventral margin concave medially, apical 1/3 portion bent dorsad, tip obtuse.	Apex acute and slightly bent dorsad, ventral margin undulating medially, and articulating with aedeagus apically.	Y-shaped
***A.chongqingana* Jiang & Yang, sp. nov.**	Entirety broad, tip convex arcuately and bent dorsally	Base with short microsetae, extending arcuately and dorsolateral posteriorly of pygofer, posterior portion with lamellar membranous structures, tip acute.	Posterior 1/2 warped dorsally, tip rounded, ventral margin articulate with paraphysis at basal 1/4 and 1/2.	Basal 1/2 stipiform, posterior 1/2 widened, tip narrowed into a cusp and curved dorsally, and articulating with aedeagus apically.	Nearly V-shaped
***A.likuni* Jiang & Yang, sp. nov.**	Basal 1/2 broad, posterior 1/2 narrow, tip warped dorsally, posterior margin truncate.	Entirety slender, arising basiventrally and extending along ventral margin of pygofer, slightly curved dorsally, median broadened with lamellar membranous structure, apical 1/3 narrow strip-shaped.	Entirety slender and straight, tip slightly bent dorsally, median and subbase concave at ventral margin.	Entirety slender and straight, posterior portion dilated, apex sharply tooth-shaped and bent dorsally, articulating apically with aedeagus at apical 2/5.	Y-shaped

### ﻿ Atkinsoniella chongqingana

Taxon classificationAnimaliaHemipteraCicadellidae

Jiang & Yang sp. nov.

A3525076-ECA7-5420-8694-84F2BB2E5CB6

https://zoobank.org/23F7EE43-2C83-44DB-A454-43F1DC100C76

Figs 1A–D , 2A–F

#### Material examined.

***Holotype***: • ♂, Wulipo National Nature Reserve, Chongqing Municipality, China, 781 m, 21 July 2021, coll. Li-Kun Zhong. ***Paratypes***: • 3 ♂♂ (light trapped), Wulipo National Nature Reserve, Chongqing Municipality, China, 790 m, 24 July 2021, coll. Li-Kun Zhong.

#### Description.

Length of male 6.9–7.3 mm. Dorsum orange. Crown with one black spot in center of vertex, and one black spot below each ocelli at basal margin; eyes black; ocellus brown; pronotum with one large inverted T-shaped black spot, and one or two black vimineous spots at each lateral margin; scutellum with one large black spot at each basal angle and connected to inverted T-shaped marking on pronotum to form seemingly lung lobe-shaped marking; forewing with black longitudinal stripe in clavus, corium, and clavus suture, respectively; posterior margin, anterior margin, and veins black, apical portion black brown, anterior marginal area black-brown in some specimens; face saffron-yellow, antennal ledge with one black spot; thorax and abdomen black in ventral view; legs brown or yellowish brown.

**Figure 1.**
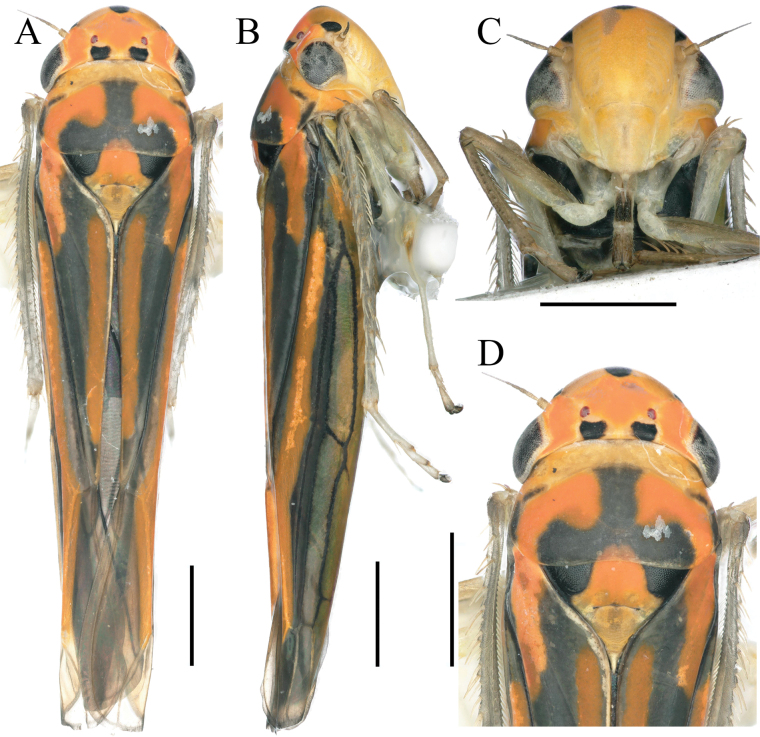
External features of *Atkinsoniellachongqingana* Jiang & Yang, sp. nov., male holotype **A** habitus, dorsal view **B** habitus, lateral view **C** face, anterior view **D** head and pronotum, dorsal view. Scale bars: 1000 μm.

Crown with anterior margin rounded and convex; crown surface flat except for lateral area of ocellus concave; ocellus located at imaginary line between anterior eye angles and tip of lateral clypeal suture; each ocellus further from other one than to adjacent eye; pronotum equal wide to head, anterior margin cambered, posterior margin slightly concave medially, lateral margins convergent anteriorly; scutellum with transverse depression slightly arcuate; face with frontoclypeus flat medially, muscle impressions distinct, clypeal sulcus slightly fuzzy medially; forewings with apical membranous area distinct and four apical cells, base of second cells more proximal than third cells transversely.

**Figure 2.**
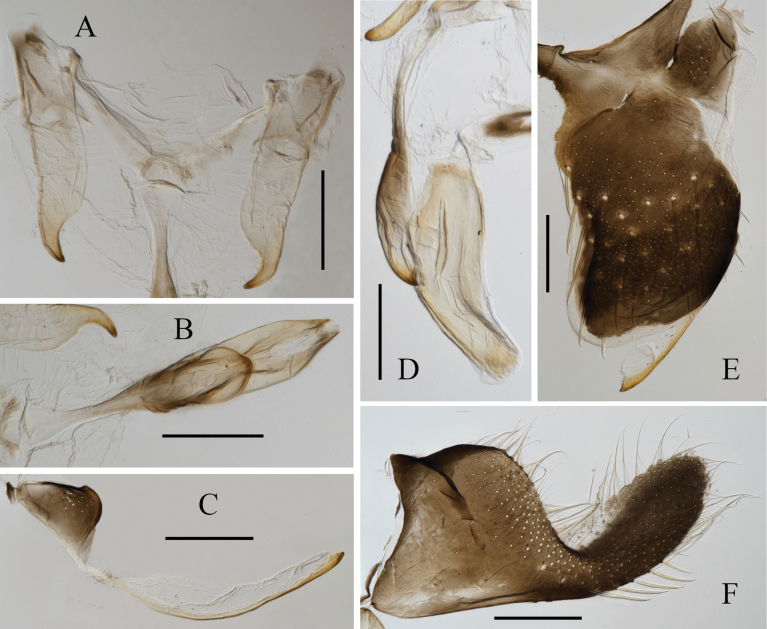
Male genitalia of *Atkinsoniellachongqingana* Jiang & Yang, sp. nov. **A** style **B** aedeagus and paraphysis, ventral view **C** pygofer process **D** aedeagus and paraphysis, lateral view **E** pygofer, lateral view **F** subgenital plate, ventral view. Scale bars: 200 μm.

Male pygofer broadly short, tip arcuately convex and bent dorsally, posterior 1/2 and median of dorsal margin with macrosetae; pygofer process with short microsetae at base, and arcuately extending dorsolateral posteriorly of pygofer, posterior portion with lamellar membranous structures, tip acute; subgenital plate broad at base, posterior 1/2 narrow and bent dorsally, with one uniseriate row of macrosetae obliquely, lateral margin and apical 1/2 with long and short microsetae; aedeagus stout, with posterior 1/2 relatively narrow and warped dorsally, tip rounded, ventral margin articulate with paraphysis at basal 1/4 and 1/2; paraphysis basal 1/2 stipiform, posterior 1/2 gradually widened, tip narrowed into cusp and dorsally curved, articulating with aedeagus apically; connective V-shaped; style broad and short, with tip acute and bent.

#### Distribution.

China (Chongqing).

#### Etymology.

The name of the new species is derived from Chongqing where the type specimens were collected.

#### Remarks.

This species is similar to *A.nigrominiatula* (Jacobi, 1944), *A.latior* Young, 1986, *A.limba* Kuoh, 1991, *A.dormana* Li, 1992, *A.divaricata* Yang, Meng & Li, 2017, *A.peaka* Yang, Meng & Li, 2017, and *A.zizhongi* Jiang & Yang, 2022 in appearance, but can be easily differentiated from these species by the following characteristics: pygofer process extending dorsolateral posteriorly of the pygofer, and its posterior portion having lamellar membranous structures; the aedeagus has its posterior 1/2 warped dorsally, and the ventral margin is articulated with the paraphysis at basal 1/4 and 1/2.

### ﻿ Atkinsoniella likuni

Taxon classificationAnimaliaHemipteraCicadellidae

Jiang & Yang sp. nov.

C2AD7E06-B77C-5681-9E05-913509095B02

https://zoobank.org/70C0BBCC-7E32-4CBF-B33E-A461F4BACAC0

Figs 3A–D , 4A–F

#### Material examined.

***Holotype***: • ♂, Wulipo National Nature Reserve, Chongqing Municipality, China, 781 m, 21 July 2021, coll. Li-Kun Zhong. ***Paratypes***: • 7 ♂♂ (light trapped)2 ♂♂, Wulipo National Nature Reserve, Chongqing Municipality, China, 781–1348 m, 18–24 July 2021, coll. Li-Kun Zhong.

#### Description.

Length of male 6.6–6.8 mm. The appearance is similar to *Atkinsoniellachongqingana* Jiang & Yang, sp. nov. Male pygofer broadly short, tip rounded and warped dorsally, median of dorsal margin and posterior 1/2 with long macrosetae; pygofer process slender and short, arising basiventrally and extending along ventral margin of pygofer, slightly curved dorsally and not as far posteriorly as pygofer apex, median lamellar broadened with membranous structure, apical 1/3 thin strip-shaped; subgenital plates basal 3/5 area broad, apical 2/5 narrow and bent dorsally, with one row of macrosetae uniseriate obliquely, long and short dense microsetae at outer lateral area of macrosetae; aedeagus slender and straight, with tip slightly bent dorsally, subbase concave at ventral margin, ventral margin articulating with dorsal margin of paraphysis medially and basally; paraphysis slender and straight, tip dilated, apex sharp teeth shaped and bent dorsally, articulating apically with aedeagus at apical 2/5 and median with aedeagus at base; connective Y-shaped; style broad at basal 2/3 and tapered at apical 1/3, apex acute and incurved.

**Figure 3.**
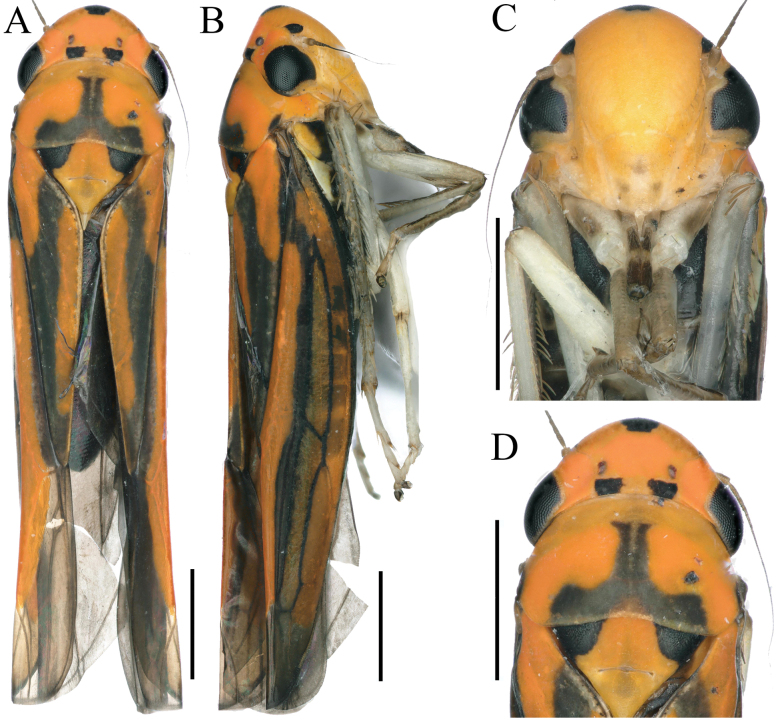
External features of *Atkinsoniellalikuni* Jiang & Yang, sp. nov., male holotype **A** habitus, dorsal view **B** habitus, lateral view **C** face, anterior view **D** head and pronotum, dorsal view. Scale bars: 1000 μm.

#### Distribution.

China (Chongqing).

#### Etymology.

The new species is named after the first name of the collector Li-Kun Zhong.

#### Remarks.

This species is similar to *A.nigrominiatula* (Jacobi, 1944), *A.latior* Young, 1986, *A.limba* Kuoh, 1991, *A.dormana* Li, 1992, *A.divaricata* Yang, Meng & Li, 2017, *A.peaka* Yang, Meng & Li, 2017, *A.zizhongi* Jiang & Yang, 2022, and *A.chongqingana* Jiang & Yang, sp. nov. in appearance, but it can be distinguished from these species by the following characteristics: (1) pygofer process smaller and not extending beyond the posterior margin of the pygofer, the median lamella is broadened with a membranous structure; (2) the aedeagus is slender and straight, its base articulating with the median of paraphysis; (3) the articulation of the aedeagus and paraphysis is located in the apical 2/5 of the aedeagus.

**Figure 4.**
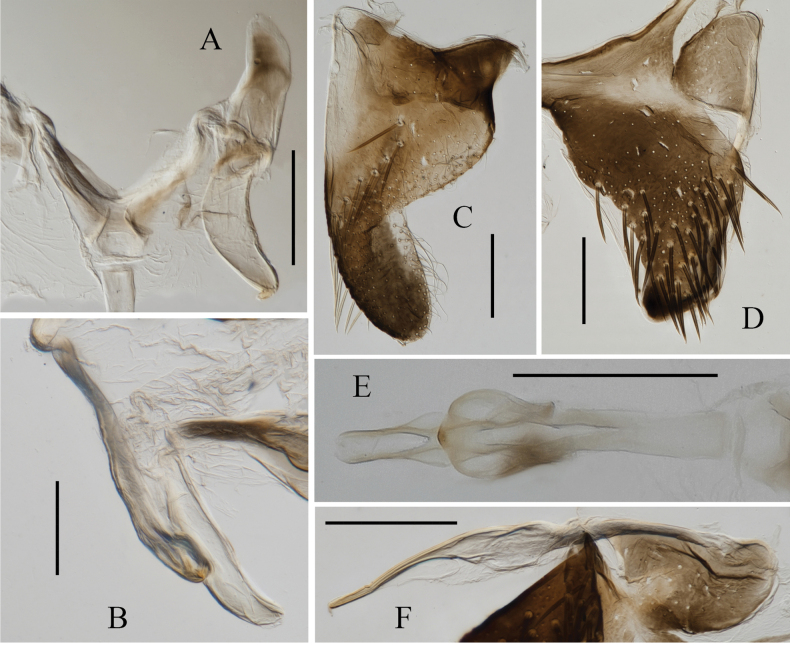
Male genitalia of *Atkinsoniellalikuni* Jiang & Yang, sp. nov. **A** style **B** aedeagus and paraphysis, lateral view **C** subgenital plate, ventral view **D** pygofer, lateral view **E** aedeagus and paraphysis, ventral view **F** pygofer process. Scale bars: 200 μm.

### ﻿ Atkinsoniella biostiolum

Taxon classificationAnimaliaHemipteraCicadellidae

Jiang & Yang sp. nov.

87AB0E32-DE7C-5F10-BA51-380665A0DE20

https://zoobank.org/4CA025A0-8798-4419-BD1D-57543DDB5D10

**Figure 5.**
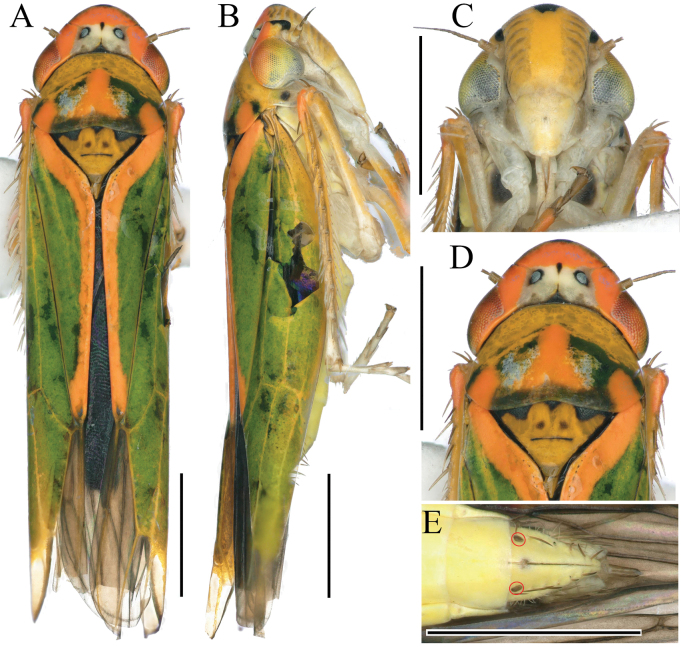
External features of *Atkinsoniellabiostiolum* Jiang & Yang, sp. nov., male holotype **A** habitus, dorsal view **B** habitus, lateral view **C** face, anterior view **D** head and pronotum, dorsal view **E** apical portion of abdomen, ventral view (red circles indicates ostioles). Scale bars: 1000 μm.

**Figure 6.**
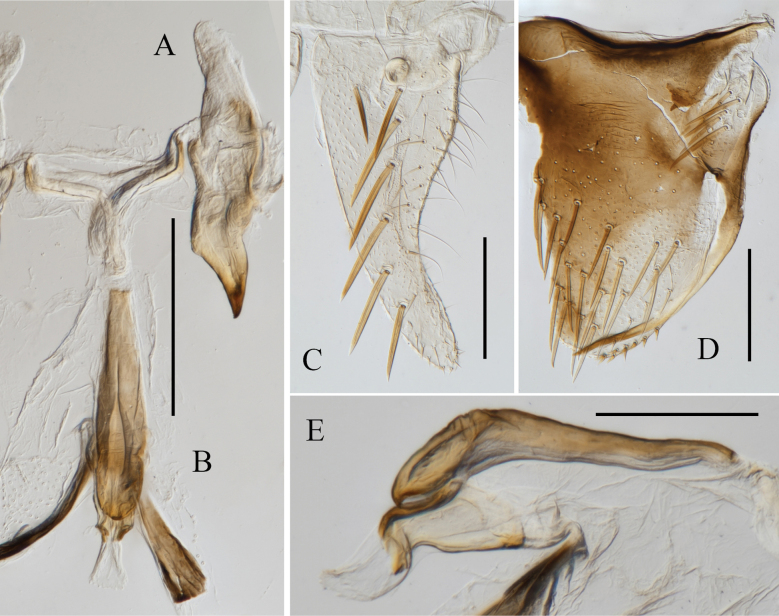
Male genitalia of *Atkinsoniellabiostiolum* Jiang & Yang, sp. nov. **A** style **B** aedeagus and paraphysis, ventral view **C** subgenital plate, ventral view **D** pygofer, lateral view **E** aedeagus and paraphysis, lateral view. Scale bars: 200 μm.

**Figure 7.**
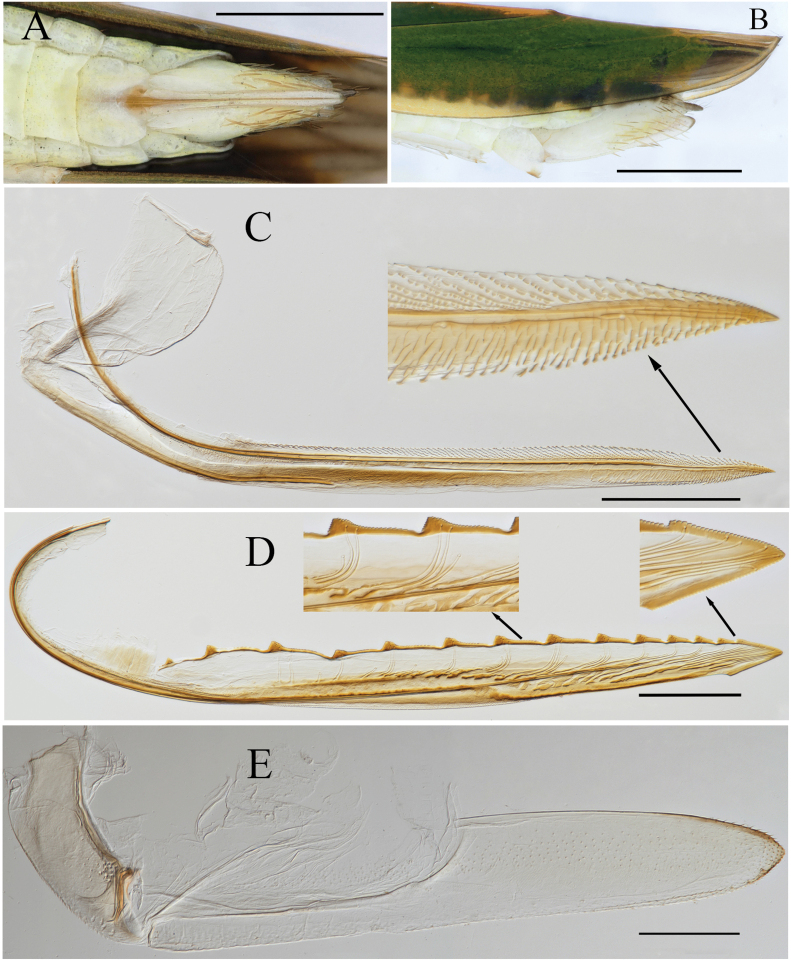
Female genitalia of *Atkinsoniellabiostiolum* Jiang & Yang, sp. nov. **A** apical portion of abdomen, ventral view **B** apical portion of abdomen, lateral view **C** first valvifer and first valvula, lateral view **D** second valvula, lateral view **E** second valvifer and gonoplac, lateral view. Scale bars: 1000 μm (**A, B**), 200 μm (**C, D, E**).

Figs 5A–E , 6A–E , 7A–E

#### Material examined.

***Holotype***: • ♂, Nongdao Town, Ruili City, Yunnan Province, China, 755 m, 4 August 2020, coll. Xian-Yi Wang. ***Paratypes***: • 1 ♂, the same data as holotype; 1 ♂ 4 ♀♀, Daweishan national forest park, Pingbian County, Honghe Hani and Yi Autonomous Prefecture, Yunnan Province, China, 1158 m, 5 June 2019, coll. Tie-Long Xu.

#### Description.

Length, male 5.3–5.4 mm, female 5.6–5.9 mm. Crown orange, posterior 1/2 with trapezoidal and yellow-white area medially, and one small drop-shaped black spot in center between ocellus, basal margin with triangular black spot medially and small triangular black brown spot below each ocellus, and coronal suture black with median discontinuous; eyes orange-black to black brown; ocelli grayish with black border distinctly; pronotum orange, with posterior 1/2 black, triangular orange macular area, concave medially, in center of black area, and one orange spot at each basal area laterally. Scutellum with three triangular black spots at basal margin and apical corner dark brown, lateral margin and transverse depression black, two small black spots above transverse depression; forewings green with yellow veins, clavus bordered with orange stripes laterally and connected with orange spots laterally on pronotum, apical membranous area black-brown; face with frontoclypeus and anteclypeus orange-yellow, muscle impressions and remaining areas dark brown, with one black spot above basal margin of antennal ledge; thorax pale yellow in ventral view, with two large black spots, legs yellow-white to gray-white, forelegs with femur and tibia orange-red, tarsus and pretarsus black-brown; abdomen yellow.

Crown with anterior margin rounded and convex; coronal suture distinct and equal to median length of crown; ocelli located slightly in front of imaginary line between anterior eye angles and tip of lateral clypeal suture, distance between ocellus equal to adjacent eye; pronotum wider than head, anterior margin rounded and convex, posterior margin with median concavity angular; scutellum with medial transverse depression slightly arcuate; forewings with distinct apical membranous area, base of second cells more proximal than third cells transversely; face with frontoclypeus flat medially, muscle impressions and clypeal sulcus blurred medially; males with one ostiole in center of base of each subgenital plate (marked by red circles in Fig. 5E).

Male pygofer broad, short, apex truncated, median of dorsal margin and posterior 1/2 with long macrosetae; pygofer process slender, arising basiventrally and extending along ventral margin of pygofer, slightly curved dorsally and just beyond pygofer apex posteriorly, median lamellar area broadened with membranous structure, apical 1/3 thin strip-shaped; subgenital plates with basal 3/5 broad, apical 2/5 narrow and bent dorsally, with one row of macrosetae uniseriate obliquely, long and short dense microsetae at outer lateral area of macrosetae; aedeagus warped medially and 8-shaped in lateral view, ventral margin articulating with dorsal margin of paraphysis medially; paraphysis slender and straight, tip dilated, apex teeth sharp and bent dorsally, articulating apically with aedeagus at apical 1/2; connective Y-shaped; style broad at basal 2/3 and tapered at apical 1/3, apex acute and incurved.

Female abdominal sternite VII, shorter than wide, posterior margin with median concavity; pygofer, in lateral view, produced posteriorly, posterior margin narrowly rounded with macrosetae at posterior portion and ventral margin; first valvifer longer than wide; first valvula apex acute, dorsal area with sculptured striae extending from basal portion of blade to apex; second valvula ventral preapical margin protruding, posterior portion arrow-shaped, blade with ~ 11 continuous large triangular teeth on expanded subapical portion and smaller teeth apically, all large teeth as well as ventral and dorsal margin of apical blade with denticles, ducts distributed in area of third teeth to apex of blade; third valvula basal 1/2 narrow and posterior 1/2 distinctly expanded, apex obtuse, and tiny setae distributed on apical portion and posterior 1/3 ventral margin of blade.

#### Distribution.

China (Yunnan).

#### Etymology.

The new species is named after the ostiole in the base of each subgenital plate.

#### Remarks.

This species can be easily differentiated from other *Atkinsoniella* species by its color, markings, characteristics of the aedeagus, especially subgenital plates with ostioles, which is the first reported characteristics in subfamily Cicadellinae.

### ﻿Key to species of *Atkinsoniella* Distant, 1908 from China (updated from Yang et al. 2017)

**Table:** 

1	Forewing completely black	***A.nigripennis* Yang & Li, 1999**
–	Forewing not black or not completely black	**2**
2	Pronotum uniform black, without distinct spots or stripes	**3**
–	Pronotum not black or not completely black	**20**
3	Apical portion of crown with a median red spot	***A.xinfengi* Yang, Meng & Li, 2017**
–	Crown without red spots	**4**
4	Forewing black with 2 brown-yellow longitudinal stripes	***A.guttata* Li, 1993 (part)**
–	Forewing black with red spots or stripes	**5**
5	Forewing with red longitudinal stripes	**6**
–	Forewing with red spots or macular area	**10**
6	Forewing with 2 red longitudinal stripes	**7**
–	Forewing with 3 red longitudinal stripes	**8**
7	Forewing with the 2 red longitudinal stripes completely disjunctive	***A.nigra* Kuoh & Cai, 1994**
–	Forewing with the 2 red longitudinal stripes connecting in the middle	***A.flavilega* Yang, Meng & Li, 2017 (part)**
8	Male head and frontoclypeus completely black	***A.nigrita* Zhang & Kuoh, 1993 (part)**
–	Male crown anterior margin and face yellow-white with black spots or stripes	**9**
9	Male vertex with a small black spot, face yellow-white without stripes	***A.nigridorsum* Kuoh & Zhuo, 1996 (part)**
–	Anterior portion of male crown with single median gray spot, frontoclypeus with black longitudinal stripe on each side	***A.fishtaila* Yang, Meng & Li, 2017**
10	Claval suture black, dividing forewing red spots or area into 2 parts	**11**
–	Claval suture black partly, forewing red spots or area complete piece	**12**
11	Forewing red area apex not exceeding the end of claval suture, frontoclypeus black	***A.lii* Yang & Zhang, 2000**
–	Forewing red area apex exceeding the end of claval suture, frontoclypeus black with a median yellow-white longitudinal stripe	***A.tuberostyla* Yang, Meng & Li, 2017**
12	Forewing red area long, apex exceeding the end of claval suture	**13**
–	Forewing red area short, apex not exceeding or only reaching the end of claval suture	**14**
13	Male pygofer process with a short horn-like branch at apical 1/3; paraphysis bifurcate at middle, clamping aedeagus	***A.nigriscens* Yang & Li, 2004**
–	Male pygofer process without branches; paraphysis not furcate with acute apex, preapical portion expanded laterally and with large dental process dorsally	***A* . *atrata* Yang, Meng & Li, 2017**
14	Male pygofer process apex fork-like, paraphysis with apex longitudinally concave medially	***A.longiaurita* Yang, Meng & Li, 2017**
–	Characters not as above	**15**
15	Apical 1/2 of male pygofer process with broad dorsal membranous lobe	***A.membrana* Yang, Meng & Li, 2017**
–	Male pygofer process smooth, without membranous lobe	**16**
16	Male pygofer process curved dorsad at base 1/3, becoming straight near apex 1/3	***A.recta* Yang, Meng & Li, 2017**
–	Male pygofer process curved not as above	**17**
17	Male pygofer process particularly long, extending posteriorly farther than apex of pygofer	***A.longa* Yang, Meng & Li, 2017**
–	Male pygofer process at most extending to apex of pygofer	**18**
18	Male pygofer posterodorsal angle finger-like, pygofer process right-angled and curved dorsad at middle	***A.rectangulata* Yang, Meng & Li, 2017**
–	Characters not as above	**19**
19	Male pygofer with apical portion raised dorsad, apicodorsal margin acute and fishtail-shaped; aedeagus apex not expanded	***A.biundulata* Meng, Yang & Ni, 2010**
–	Male pygofer with apical portion produced round-horned; aedeagus apex expanded	***A.expanda* Yang, Meng & Li, 2017**
20	Forewing without distinct spots or stripes	**21**
–	Forewing with distinct spots or stripes	**53**
21	Forewing black-brown, the joint area of 2 forewings orange-red, veins of costal margin and corium orange	***A.xanthovena* Yang & Li, 2002**
–	Characters of forewing not as above	**22**
22	Forewing of living body cyan, exsiccatae yellow-brown, forewing hyaline next to costal margin	***A.variata* Young, 1986**
–	Characters of forewing not as above	**23**
23	Pronotum with distinct spots or stripes	**24**
–	Pronotum without distinct spots or stripes	**27**
24	Scutellum tawny or orange, with basal black side spots	**25**
–	Scutellum black completely	**26**
25	Scutellum tawny, apex with a black spot	***A.sulphurata* (Distant, 1908) (part)**
–	Scutellum orange, apex without black spots	***A.longiuscula* Feng & Zhang, 2015 (part)**
26	Crown black, anterior 1/2 with a median yellow-white spot; inside eyes yellowish-white, region broad; male pygofer posterodorsal margin finger-like	***A.fuscopenna* Yang & Li, 2004**
–	Crown black, anterior 1/2 without median yellow-white spots, inside eyes yellowish white, region narrow; male pygofer posterodorsal margin angular	***A.guttata* Kuoh, 1992 (part)**
27	Basal portion of crown without median black spots	**28**
–	Basal portion of crown with single median black spot	**31**
28	Male pygofer process with apical 1/2 straightened posteriorly, exceeding apical margin of pygofer, without branch	***A.uniguttata* Li, 1993**
–	Male pygofer process with small branch at subapex	**29**
29	Apical portion of crown with large, median black squared spot; aedeagus broad and short, apex truncated	***A.cyclops* (Melichar, 1914)**
–	Apical portion of crown without black spots or only with a minimal spot; aedeagus not as above	**30**
30	Apical portion of crown without black spots, face yellowish brown, frontoclypeus with a median yellow-white longitudinal stripe; aedeagus with finger-like rounded tip	***A.duna* Yang, Meng & Li, 2017**
–	Apical portion of crown without black spots or only with a minimal spot, face uniform yellowish white; aedeagus with acute tip not finger-like	***A.xanthoabdomena* Yang, Meng & Li, 2017**
31	Crown with 2 black, median, parallel rhomboid spots	***A.rhomboida* Yang, Meng & Li, 2017**
–	Crown with none or 1 median black spot	**32**
32	Apical portion of crown without black spots, the spot of basal portion V-shaped	***A.curvata* Yang & Li, 1980**
–	Apical portion of crown with median black spot, spot of basal portion not V-shaped	**33**
33	Crown with large basal black spot, distinctly larger than anterior median one	**34**
–	Crown with small basal black spot, as large as or smaller than anterior median	**35**
34	Scutellum without black spots, forewing orange, subapical region orange-red or red	***A.wui* Yang, Meng & Li, 2017**
–	Scutellum with a black spot in each basal angle, forewing ivory	***A.albipenna* Yang, Meng & Li, 2017**
35	Forewing ivory, base orange-red or red	**36**
–	Forewing orange, yellow-green, gray-brown, or red-brown except apical membrane	**37**
36	Abdominal venter black completely; male subgenital plate without macrosetae	***A.punica* Yang & Li, 2002**
–	Abdomen yellow, or yellow-brown, or only with black apex; male subgenital plate with uniseriate macrosetae	***A.longiuscula* Feng & Zhang, 2015 (part)**
37	Mesothethium with 2 large black or black-brown spots	**38**
–	Mesothethium without spots or stripes	**44**
38	Scutellum without black spots	**39**
–	Scutellum with a black spot in each basal angle	**40**
39	Male pygofer process slender; aedeagus narrowed to the end, apex acute	***A.multiseta* Yang, Meng & Li, 2017**
–	Male pygofer process broad and long, tapering apically and bending inside; aedeagus with approximately parallel sides, apex round	***A.changae* Yang, Meng & Li, 2017**
40	Forewing gray-brown; male pygofer process with a small branch subapically	***A.zhangmuensis* Yang, Meng & Li, 2017**
–	Forewing orange; male pygofer process without branches	**41**
41	Male pygofer process with apical 1/3 constricted and contorted	**42**
–	Male pygofer process normal, not constricted or contorted	**43**
42	Apical 1/3 portion of male pygofer process willow-leaf-shaped, straight; aedeagus broad and short	***A.flavipenna* Li & Wang, 1992**
–	Apical 1/3 portion of male pygofer process sickle-shaped; aedeagus slender	***A* . *liui* Yang, Meng & Li, 2017**
43	Frontoclypeus with a median thin tumor near apex; whole paraphysis curved dorsad	***A.rinkihonis* (Matsumura, 1912)**
–	Frontoclypeus without the tumors; paraphysis arched, apical 1/2 right-angled curved dorsad	***A.bowa* Yang, Meng & Li, 2017**
44	Male pygofer process with branch or toothed process subapically	**45**
–	Male pygofer process without branch or process	**47**
45	Male pygofer process with membranous branch; paraphysis curved dorsad from median portion, and with apical 1/3 curved posteroventrally	***A.yunnanana* Yang, Meng & Li, 2017**
–	Male pygofer process with toothed process subapically, paraphysis curved not as above	**46**
46	Male pygofer with posterodorsal margin arc-shaped, apex of pygofer process thick; aedeagus slender, apical 1/2 straight	***A.thaloidea* Young, 1986**
–	Male pygofer process with posterodorsal margin roundly angular, apex of pygofer process thin; aedeagus stout, apex curved dorsad	***A.thalia* (Distant, 1918)**
47	Male pygofer truncated apically; pygofer process contorted and curved medially	***A.aurantiaca* Cai & Kuoh, 1995**
–	Male pygofer rounded apically; pygofer process not contorted or curved medially	**48**
48	Scutellum without spots and stripes	**49**
–	Scutellum with black or black-brown spot in each basal angle	**51**
49	Male pygofer process lamellate, posterior 1/3 portion broadly lamellate and twisted backwards, apex acute; connective V-shaped	***A.wangi* Jiang & Yang, 2023**
–	Male pygofer process slender, posterior 1/3 not as above; connective Y-shaped	**50**
50	Male aedeagus with posterior portion angular	***A.warpa* Yang, Meng & Li, 2017**
–	Male aedeagus with posterior margin truncate	***A.stenopyga* Jiang & Yang, 2023**
51	Head, thorax, and base of forewing ivory in dorsal view; male pygofer process broad and flat, apex beak-shaped, abruptly acute and slightly curved; aedeagus slightly curved dorsad medially	***A.beaka* Yang, Meng & Li, 2017**
–	Head, thorax, and forewing orange-yellow or gray-brown in dorsal view; male pygofer process slender, apex not beak-shaped; aedeagus straight	**52**
52	Head, thorax, and forewing gray-brown in dorsal view; male pygofer nearly truncated apically, apical margin with minute dents, apical 1/3 portion of pygofer process abruptly narrowed; aedeagus with ventral tumor medially	***A.heae* Yang, Meng & Li, 2017**
–	Head, thorax, and forewing orange-yellow in dorsal view; male pygofer with apex roundedly angular dorsad, apical margin smooth, pygofer process tapering to the end; aedeagus without ventral tumor	***A.tiani* Yang, Meng & Li, 2017**
53	Forewing dark yellow-brown, with muddy yellow or gray-white transparent or translucent spots	***A.huangi* Yang & Zhang, 2000**
–	Forewing not as above	**54**
54	Forewing black, with 3 longitudinal grayish white stripes; pronotum with 2 small black spots abreast in the center, and posterior area with 2 large black spots transversely	***A.yingjiangensis* Jiang & Yang, 2023**
–	Forewing and pronotum not as above	**55**
55	Forewing green, clavus bordered with orange stripes laterally, males with 1 ostiole in the center of the base of each subgenital plate	***A.biostiolum* Jiang & Yang, sp. nov.**
–	Forewing and subgenital plates not as above	**56**
56	Forewing white or gray-white, with brown or black-brown stripes	**57**
–	Forewing black with red or brown-yellow stripes, or forewing brown-yellow with black or yellow-brown stripes	**58**
57	Basal portion of crown with 工-shaped black stripe medially; forewing gray-white, costal margin, inner margin, and veins black-brown, broad longitudinal brown stripe along claval suture	***A.motuoensis* Meng, Yang & Ni, 2010**
–	Basal portion of crown with large black spot medially; forewing ivory, costal and inner margins black-brown, with longitudinal black-brown stripe parallel to costal and inner margins	***A.alcmena* (Distant, 1908)**
58	Scutellum completely black	**59**
–	Scutellum not completely black	**85**
59	Pronotum with 2 large white spots; forewing clavus with broad longitudinal white strip	***A.albimacula* Yang & Li, 2002**
–	Characters not as above	**60**
60	Forewing with red stripes or spots	**61**
–	Forewing without red stripes or spots	**81**
61	Forewing clavus base with red spot or short stripe, costal margin with or without orange-red stripe	***A.alternata* Young, 1986**
–	Forewing with red stripes not as above	**62**
62	Forewing with 2 longitudinal red stripes	**63**
–	Forewing with 3 longitudinal red stripes	**72**
63	Red stripes of forewing short, extending only to end of claval suture	***A.furipygofera* Yang & Meng, 2011**
–	Red stripes of forewing long, extending farther than end of claval suture	**64**
64	Forewing with the 2 longitudinal red stripes connecting in the middle	***A.flavilega* Yang, Meng & Li, 2017(part)**
–	Forewing with the 2 longitudinal red stripes completely separated	**65**
65	Male paraphysis with 3 toothed processes apically	***A.tridentata* Yang & Li, 2011**
–	Male paraphysis with single pointed process apically	**66**
66	Head and thorax with many small scattered red spots; caudodorsal margin of male pygofer not produced in angular or flat finger-shaped process	***A.rufistigma* Yang, Meng & Li, 2017 (part)**
–	Head and thorax without small red spots; caudodorsal margin of male pygofer produced in angular or flat finger-shaped process	**67**
67	Caudodorsal margin of male pygofer extended posteriorly forming angular process	**68**
–	Caudodorsal margin of male pygofer extended posteriorly forming flat finger-shaped process	**70**
68	Basal portion of crown with transverse black band; pronotum with 2 transverse red stripes medially; male pygofer process with flat angular process medially	***A.zaihuai* Yang & Meng, 2011**
–	Basal portion of crown without black strip, but middle portion of crown with 2 longitudinal black stripes across ocelli; pronotum with 2 large orange-red spots; male pygofer process without flat angular process	**69**
69	Face orange-red, frontoclypeus with small black spot medially, clypeal suture with triangular black spot; male pygofer process with apical 1/3 flat and broad, aedeagus slender	***A.angula* Kuoh, 1992**
–	Face black, frontoclypeus with a longitudinal orange-red stripe medially, anteclypeus with lateral orange-red spot, gena and maxillary plate yellow-white; male pygofer process strip-shaped, apical 1/2 tapering, aedeagus stout	***A.nigrosteaka* Li & Wang, 1994**
70	Crown black except muddy yellow posterolateral margin; red band of pronotum short and narrow	***A.xanthonota* Kuoh, 1994**
–	Crown muddy yellow or orange-red, basal and apical portion with black stripes; red band of pronotum long, width variable	**71**
71	Crown with 3 black spots, 1 at vertex, 2 under ocelli; red band of pronotum broad and long	***A.grahami* Young, 1986 (part)**
–	Crown with irregular transverse black bands on apical and basal portion; red band of pronotum slender	***A.rubrostriata* Kuoh, 1992**
72	Pronotum with an uninterrupted median transverse red band, face yellow-white without stripe	***A.nigridorsum* Kuoh & Zhuo, 1996 (part)**
–	Characters not as above	**73**
73	Frontoclypeus and anteclypeus black completely	**74**
–	Frontoclypeus and anteclypeus not all black	**76**
74	Median portion of pronotum with a transverse red band or several continuous red spots; red stripes of forewing broad	***A.transifasciata* Yang, Meng & Li, 2017**
–	Median portion of pronotum with 2 red spots or oblique stripes; red stripes of forewing narrow and thin	**75**
75	Two red longitudinal stripes on corium of forewing disjunctive completely, legs yellow-white	***A.yani* Yang, Meng & Li, 2017**
–	Two red longitudinal stripes on corium of forewing joined at base, legs black	***A.insignata* (Haupt, 1924)**
76	Pronotum with 2 rounded red spots, frontoclypeus completely black	***A.nigrita* Zhang & Kuoh, 1993 (part)**
–	Pronotum with red spots not rounded, frontoclypeus not completely black	**77**
77	Crown with a large fork-shaped black spot on basal-median portion; frontoclypeus with a large lateral black spot; anteclypeus with a long median black stripe	***A.contrariuscula* (Jacobi, 1944)**
–	Characters not as above	**78**
78	Pronotum with a transverse red stripe medially	***A.mediofasciola* Yang & Li, 2002**
–	Pronotum with 2 transverse red stripes medially	**79**
79	Crown completely black	***A.goosenecka* Yang, Meng & Li, 2017**
–	Crown orange-red with black spots	**80**
80	Face yellow-white, but frontoclypeus with basal margin black, basal 1/2 with lateral longitudinal black stripe, whole inverted U-shaped	***A.hupehna* Young, 1986**
–	Face orange-red, with Y-shaped or T-shaped black stripes	***A.dactylia* Yang & Li, 2000**
81	Forewing yellow-green, costal and inner margins black, corium with 2 black spots	***A.nigricephala* Li, 1992**
–	Forewing black with orange stripes	**82**
82	Forewing with 4 large orange spots	***A.dubia* Young, 1986**
–	Forewing with 2 longitudinal orange or yellow-brown stripes	**83**
83	Crown yellow-brown, with 2 Y-shaped black stripes medially; face with lorum completely black	***A.flexa* Kuoh, 1992**
–	Crown with stripes not as above; face with lorum orange-yellow or only basal portion black	**84**
84	Crown black except yellow-white posterolateral margin and inner side of eyes; anteclypeus black, lorum black basally	***A.guttata* Kuoh, 1992 (part)**
–	Crown orange-red or orange-yellow, with 3 distinct black spots, 1 at vertex, 2 under ocelli; anteclypeus with a black spot basally, lorum orange-yellow	***A.grahami* Young, 1986 (part)**
85	Scutellum black with red spots	**86**
–	Scutellum without red spots	**87**
86	Pronotum bright red, anterior margin with a large median black spot, posterior margin with black boundary, median portion with or without black stripe linked; scutellum with a large red spot medially	***A.brevistyla* Yang & Li, 2004**
–	Pronotum black, anterior margin with 2 transverse red stripes or spots, median portion with 2 long transverse red stripes; scutellum with 2 or 4 red spots	***A.rufistigma* Yang, Meng & Li, 2017 (part)**
87	Forewing with red stripes	**88**
–	Forewing without red stripes	**101**
88	Pronotum date red, with 3 black spots arranged in regular triangle	***A.trimaculata* Li, 1992**
–	Characters not as above	**89**
89	Forewing with 3 longitudinal red stripes	**90**
–	Forewing with more than 3 red stripes	**93**
90	Male pygofer constricted medially, apex arrow-shaped	***A.cuspidata* Meng, Yang & Ni, 2010 (part)**
–	Characters not as above	**91**
91	Male pygofer process with thumb-shaped dorsal projection subapically	***A.jini* Yang, Meng & Li, 2017**
–	Male pygofer process without any projections	**92**
92	Male pygofer process with membranous lobe at apicodorsal margin; paraphysis narrow and straight, apex expanded and curved sickle-shaped, ventral margin deeply concave medially	***A.nigrisigna* Li, 1992 (part)**
–	Male pygofer process without membranous lobe, paraphysis with apical portion curved dorsad, apex acute	***A.heiyuana* Li, 1992**
93	Male paraphysis forked apically and clamping median of aedeagus	***A.divaricata* Yang, Meng & Li, 2017**
–	Male paraphysis not forked apically	**94**
94	Male pygofer process extending posterodorsally, then posteriorly attenuated and sharply curved apically	***A.latior* Young, 1986**
–	Characters not as above	**95**
95	Aedeagus with paired flat dorsal triangular processes at middle	***A.dormana* Li, 1992**
–	Aedeagus with dorsal aspect straight	**96**
96	Apical portion of male pygofer peaked; apex of paraphysis extending posteriorly nearly to apex of aedeagus	***A.peaka* Yang, Meng & Li, 2017**
–	Apical portion of male pygofer not peaked; apex of paraphysis extending posteriorly only to middle of aedeagus	**97**
97	Dorsal margin of male pygofer with an angular flat process near base, aedeagus long, paraphysis with lateral produced flatly subapically	***A.limba* Kuoh, 1991**
–	Characteristics not as above	**98**
98	Dorsal margin of male pygofer straight, aedeagus short and dorsal margin near straight, paraphysis with apex tapered	***A* . *nigrominiatula* (Jacobi, 1944)**
–	Characteristics not as above	**99**
99	Aedeagus stout with posterior 1/2 relatively narrow and warped dorsally, aedeagus 2× wider than paraphysis in lateral view	***A* . *chongqingana* Jiang & Yang, sp. nov.**
–	Aedeagus slender, aedeagus and paraphysis subequal in width in lateral view	**100**
100	Male pygofer process median lamellar broadened with membranous structure, apical 1/3 thin strip-shaped, acute at apex; paraphysis articulating apically with aedeagus at apical 2/5	***A* . *likuni* Jiang & Yang, sp. nov.**
–	Male pygofer process dorsad membranous transparent apically, not acute at the apex; paraphysis articulating apically with aedeagus at apical 1/2	***A* . *zizhongi* Jiang & Yang, 2022**
101	Forewing orange-yellow or brown-yellow, with black spots	**102**
–	Forewing black, with longitudinal orange-yellow stripes; or orange-yellow with black stripes	**103**
102	Apical margin of crown with black spot medially; forewing with 4 large black spots, 1 located at subbasal portion of clavus, other 3 located at subbasal, median, and subapical portions of corium, respectively	***A.sulphurata* (Distant, 1908) (part)**
–	Apical 1/2 of crown with a shawl-shaped black stripe; forewing with 3 black spots, 1 located at apical portion of clavus, others located at basal 1/3 and 2/3 portions of corium, respectively	***A.malaisei* Young, 1986 (part)**
103	Forewing orange-yellow, with a longitudinal black stripe medially	**104**
–	Characters not as above	**105**
104	Apical margin of crown with a black spot medially	***A.sulphurata* (Distant, 1908) (part)**
–	Apical 1/2 of crown with a shawl-shaped black stripe	***A.malaisei* Young, 1986 (part)**
105	Forewing with 2 longitudinal muddy-yellow stripes	***A.xanthovitta* Kuoh, 1994**
–	Forewing with > 2 longitudinal stripes	**106**
106	Body smaller, length < 5.5 mm; clavus orange-yellow, with a longitudinal black stripe medially	***A.opponens* (Walker, 1851)**
–	Body larger, length > 6 mm; clavus orange-yellow, without longitudinal black stripes medially	**107**
107	Male pygofer constricted medially, apex arrow-shaped	***A.cuspidata* Meng, Yang & Ni, 2010 (part)**
–	Male pygofer with apex curved dorsally, apical margin roundly produced	***A.nigrisigna* Li, 1992 (part)**

## ﻿Discussion

Currently, the identification of Cicadellinae species is mainly based on their external morphology and male genitalia characteristics of adults. However, there are some species that exhibit similar external morphologies, but the characteristics of the male genitalia are obviously different, or the characteristics of male genitalia are similar, but external morphologies are different. These situations make the identification of some Cicadellinae species difficult at species level, especially for the female specimens. *Atkinsoniellanigrominiatula* (Jacobi, 1944), *A.latior* Young, 1986, *A.limba* Kuoh, 1991, *A.dormana* Li, 1992, *A.divaricata* Yang, Meng & Li, 2017, *A.peaka* Yang, Meng & Li, 2017, *A.zizhongi* Jiang & Yang, 2022, *A.chongqingana* Jiang & Yang, sp. nov., and *A.likuni* Jiang & Yang, sp. nov. are similar in appearance but differ in their male genitalia. As the characteristics of female genitalia of subfamily Cicadellinae species are not obvious, those of the female specimens of *A.chongqingana* Jiang & Yang, sp. nov. and *A.likuni* Jiang & Yang, sp. nov. cannot be provided as the females have the same appearance, making their identification confusing; therefore, molecular methods are necessary to help solve these difficulties and provide more accurate species delimitations. In addition, the investigations into the biology and ecology may be good directions for better understanding the known and newly described leafhopper species in the future.

## Supplementary Material

XML Treatment for Atkinsoniella

XML Treatment for Atkinsoniella chongqingana

XML Treatment for Atkinsoniella likuni

XML Treatment for Atkinsoniella biostiolum

## ﻿Additional information

### Author ORCIDs

Yan Jiang https://orcid.org/0000-0002-8980-5344

Xiao-Fei Yu https://orcid.org/0000-0002-1362-9524

Mao-Fa Yang https://orcid.org/0000-0002-5523-6825

### Data availability

All of the data that support the findings of this study are available in the main text.
